# Early Diagnosis of HIV among Infants Born to HIV-Positive Mothers on Option-B Plus in Kampala, Uganda

**DOI:** 10.1155/2017/4654763

**Published:** 2017-10-19

**Authors:** Jonathan Izudi, Sylvia Auma, John Bosco Alege

**Affiliations:** ^1^Department of Community Health, Faculty of Medicine, Mbarara University of Science and Technology, Mbarara, Uganda; ^2^Institute of Public Health and Management, International Health Sciences University, Kampala, Uganda

## Abstract

**Introduction:**

Globally, there is delay in accessing early HIV diagnosis (EID) among HIV exposed infants (HEIs). With paucity of data on EID use at Kisenyi Health Center, this study assessed factors associated with EID use among HEIs (HIV exposed infants).

**Method:**

This was a cross-sectional study of 246 HIV-positive mother-baby pairs. Data was collected by structured questionnaire, double-entered in EpiData, and analyzed with STATA using multinomial logistic regression at 5% significance level.

**Results:**

132 (53.7%) HEIs were not tested, 60 (24.4%) tested outside EID guideline, and 54 (21.9%) tested per the guideline. Testing per guideline was associated with maternal age above 30 years (AOR = 2.75; 95% CI: 1.20–6.34; *P* = 0.017); testing outside the guideline was associated with maternal HIV serostatus disclosure (AOR = 2.70; 95% CI: 1.10–6.63; *P* = 0.003) and four or more antenatal care (ANC) visits (AOR = 3.25; 95% CI: 1.23–8.59; *P* = 0.017). However, maternal knowledge of HIV transmission was associated with testing outside the guideline (AOR = 2.90; 95% CI: 1.10–7.65; *P* = 0.032) and per the guideline (AOR = 3.70; 95% CI: 1.39–9.88; *P* = 0.009).

**Conclusion:**

Timely EID testing was low. Improving maternal knowledge of EID during ANC visits and positive living empowerment is critical.

## 1. Background

Without timely access to ART (antiretroviral therapy), 20% of HIV- (Human Immunodeficiency Virus-) positive infants will die before 6 months [1], 35–40% will die before 1 year [1, 2], and over 50% will die by 2 years [3]. Early diagnosis of HIV (EID) is hence fundamental in early identification of HIV-positive infants and subsequent ART initiation. However, timely receipt of EID remains a challenge in most countries despite a 10-fold increase and improved access to ART between 2005 and 2015 [4]. In addition, the proportion of HEIs with timely access to EID varies between 30% and 90% in many countries [4]. In sub-Saharan Africa, about 75% of HEIs have delayed access to EID services resulting into substantial mortality [5]. With delayed access to EID, current progress towards zero new HIV infections and starting 90% or more HIV-positive infants on ART might be reversed [6]. EID thus facilitates timely access to ART and in so doing, it optimize viral load suppression, reduce HIV related morbidity and mortality, and improve the overall quality of life of HIV-positive children. Evidence indicates that timely initiation of ART reduces the risk of infant mortality by 76% within the first 3 months of life [7, 8] and the progression to AIDS (Acquired Immunodeficiency Syndrome), an advanced stage of HIV infection, by three quarters [8].

In June 2012, Uganda's Ministry of Health adopted Option-B plus, a strategy of starting all HIV-positive pregnant and lactating mothers on ART irrespective of their clinical and immunological stage in order to eliminate mother to child transmission of HIV (EMTCT) [9].

Via a provider initiated HIV testing and counseling (PITC), all pregnant mothers receive HIV testing during antenatal care (ANC) visits, labor, and delivery.

However, postpartum mothers receive HIV testing during postnatal-care visits. When found HIV-positive, pregnant and lactating mothers are started on ART and cotrimoxazole prophylaxis, and the viral load is monitored for response to ART [9]. From birth to 18 months, HEIs undergo routine EID care. At birth, nevirapine syrup is introduced but the dose is adjusted according to weight and age bands until 6 weeks. At 6 weeks, nevirapine syrup is stopped, the first HIV test using Deoxyribonucleic Acid-Polymerase Chain Reaction (DNA-PCR) is performed, and cotrimoxazole prophylaxis is introduced (also adjusted according to age and weight bands) until 18 months. Exactly 6 weeks after breastfeeding cessation, the second DNA-PCR test is performed and at 18 months, a final HIV antibody test is done. Should a HEI test HIV-positive at any time point, ART is initiated alongside cotrimoxazole prophylaxis. Regarding breastfeeding, HEIs exclusively breastfeed for the first 6 months before commencing complementary feeding, and breastfeeding ceases at 1 year [6, 10]. Despite these recommendations, merely two in every five HEIs access EID by DNA-PCR tests within two months of birth (first DNA-PCR) in Uganda [11].

At Kisenyi HC (Health Center) IV, routine data indicates that a substantial number of HEIs do not receive EID by DNA-PCR test at 6 weeks. First, this presents a missed opportunity for early HIV diagnosis and ART initiation. Second, the absence of information on factors associated with receipt of DNA-PCR to inform EID programming is a big hindrance to improving EID services. In this study, we investigated factors associated with use of EID among HEIs receiving care at Kisenyi HCIV in Kampala district, Uganda.

## 2. Method

This study was conducted at Kisenyi HCIV, one of the two health facilities in Kampala Central Division, Kampala district. Kisenyi is a slum setting in the middle of down town Kampala where a mix of numerous languages in Uganda, Eastern Congo, Tanzania, Rwanda, Kenya, Somalia, and Ethiopia are spoken [12]. The study setting, Kisenyi HCIV, lies in Central Kampala along Mwanga II Road. The health facility is managed by Kampala Capital City Authority (KCCA), a new administrative body assigned with the role of managing health services delivery in Kampala City. The authority controls five divisions of Kampala Central, Nakawa, Lubaga, Makindye, and Kawempe. Each division has two health centers apart from Nakawa. In total, there are nine health centers and two hospitals [13]. According to the Uganda Bureau of Statistics 2014 data, there are 1,516,210 persons in Kampala district [14].

We used an analytical cross-sectional design to describe and analyze factors associated with use of EID among HEIs. The respondents were HIV-positive postpartum mothers or caregivers of HEIs in the EID program of Kisenyi HCIV, selected purposively. Using Kish and Leslie's formula [15], 246 respondents within 95% confidence limit, 5% precision, and 20% estimated proportion of EID use at 6 weeks were sampled and interviewed. Since we aimed to collect data over a 20-day period, first, the sample size was divided by the total number of days for data collection to get the average number of respondents to interview per day. We then used systematic sampling to establish a sampling interval by dividing the total number of expected HIV-positive mothers or caregivers on a particular clinic day by the total number of respondents required for daily interviews.

From the sampling interval, a convenience sampling method was employed to select respondents at a time until the total sample size was reached.

Prior to data collection, all data collection tools were forward and backward translated and pretested outside the study area. The data was collected by trained and supervised Research Assistants using a structured questionnaire administered by face to face interviews between December 2016 and April 2017. All interviews were conducted in a quiet room within the mother-baby care point, a clinic within the EID program where HIV-positive mothers/caregivers and HEIs are paired and followed until 18 months. Interviews were conducted from Monday to Friday between 8.30 a.m. and 12.00 p.m. All completed questionnaires were reviewed in real time for completeness and accuracy by a research team leader.

Data was collected on use of EID, maternal, and health systems-related factors. Maternal age and employment status, level of education, knowledge on HIV transmission and prevention during pregnancy, labor and delivery, distance to the nearest health facility to access EID services, ever receiving health education on HIV transmission, and knowledge of DNA-PCR test frequency and timing were variables on which data was collected. Data on use of EID was verified using EID clinical charts and registers.

Collected data was double-entered in EpiData version 3.1 (EpiData Association, Odense, Denmark) [16], checked for data quality, and exported to STATA Version 12 (StataCorp, College Station, TX, USA) for univariate, bivariate, and multivariate analysis at 5% significance level. Frequencies and percentages were calculated for categorical variables and measures of central tendency for continuous variables.

The dependent variable, EID use, was defined by three outcomes: HEIs not tested by DNA-PCR, HEIs tested by DNA-PCR outside EID guideline (early and late), and HEIs tested by DNA-PCR per EID guideline (between 6 and 8 weeks). DNA-PCR test performed before 6 weeks was considered early and that performed after 8 weeks was regarded late. The denominator was the sample size.

Tests of associations were performed using the Chi-squared test for large cell counts (when the cross-tabulations had all cell counts equal or above five) or the Fisher's exact test (when the cross-tabulations had any cell count less than five) for categorical independent variables and the *F*-test for continuous variables. Test of collinearity between variables was performed, and variables with variance inflation factor (VIF) greater than ten were excluded in the final model. Since the outcome variable was nominal, a multinomial logistic regression analysis was performed to determine factors independently associated with use of EID, with HEIs not tested by DNA-PCR being the reference category. The output of multinomial logistic regression analysis was stated in odds ratios with corresponding 95% confidence intervals (CI) and probability values (*P* values).

This study was approved by the Research Ethics Committee of International Health Sciences University and the Health Department of Kampala Capital City Authority (KCCA). All respondents gave a written or thumb printed informed consent before participation.

## 3. Results

### 3.1. Sociodemographic Characteristics of Respondents and Use of EID

The mean age of mothers with HEIs not tested for HIV by DNA-PCR was 25.5 ± 6.12 years, that for mothers with HEIs tested outside the EID guideline was 29.3 ± 5.35 years, and that for mothers with HEIs tested per the EID guideline was 29.69 ± 5.69 years (*P* < 0.0001).

Among 163 mothers below the age of 30 years, 103 (63.2%) HEIs were not tested by DNA-PCR, 34 (20.9%) were tested outside the EID guideline, and 26 (16.0%) were tested according to the guideline. In contrast, among 83 mothers above 30 years of age, 29 (34.9%) HEIs were never tested, 26 (31.3%) were tested outside the guideline, and only 18 (33.7%) were tested per the guideline (*P* < 0.001) (Table 1).

Of 43 mothers without formal education, 31 (72.1%) HEIs were not tested, nine (20.9%) were tested outside the EID guideline, and merely three (7.0%) were tested per the guideline. Compared to 55 mothers that completed primary education, the corresponding numbers were 44 (80.0%), six (10.9%), and five (9.1%). Similarly, of 146 mothers that completed secondary education and beyond, the respective figures were 57 (38.5%), 45 (30.4%), and 46 (31.1%) (*P* < 0.001).

Regarding marital status of the mothers, 167 (67.9%) were single/unmarried while 79 (32.1%) were married. Among single/unmarried mothers, 101 (60.5%) HEIs were not tested, 35 (21.0%) were tested outside the EID guideline, and 31 (18.6%) were tested per the guideline. For married mothers, the respective figures were 31 (39.2%), 25 (31.7%), and 23 (29.1%) (*P* = 0.008).

In terms of employment, 115 (46.7%) of the mothers were unemployed, 26 (10.6%) had formal employment, and 105 (42.7%) were self-employed. Among unemployed mothers, only 14 (12.2%) HEIs were tested per the EID guideline, 20 (17.4%) were tested outside the guideline, and 81 (70.4%) were not tested. For mothers with formal employment, only two (7.7%) HEIs were not tested, 13 (50.0%) were tested outside the EID guideline, and 11 (42.3%) were tested per the guideline. Among self-employed mothers, 49 (46.7%) HEIs were not tested, 27 (25.7%) were tested outside the EID guideline, and only 29 (27.6%) were tested per the guideline (*P* < 0.001).

118 (68.2%) HEIs were not tested, 31 (17.9%) were tested outside EID guideline, and 24 (13.9%) were tested per the guideline among 173 mothers that had no knowledge of HIV transmission during pregnancy, labor and delivery, and breastfeeding. The equivalent proportion among 73 mothers with this knowledge was 14 (19.2%), 29 (39.7%), and 30 (41.1%), respectively (*P* < 0.001).

Of 199 mothers without knowledge of the correct number of times and schedules for EID testing, 123 (61.8%) HEIs were not tested, 41 (20.6%) were tested outside EID guideline, and 35 (17.6%) were tested per the guideline. Likewise, of 47 mothers with the knowledge, nine (19.2%) HEIs were not tested, 19 (40.4%) were tested outside the guideline, and another 19 (40.4%) were tested per the guideline (*P* < 0.001).

Of 118 mothers with undisclosed HIV serostatus (to a spouse, relative, friend, or significant others), 116 (64.1%) HEIs were not tested, 32 (17.7%) were tested outside the EID guideline, and 35 (17.6%) were tested per the guideline. Conversely, among 65 mothers with disclosed HIV serostatus, the proportion of testing was 16 (24.6%), 28 (43.1%), and 21 (32.3%), respectively (*P* < 0.001).

Among 135 mothers that attended less than four ANC visits during the recent pregnancy, 85 (63.0%) HEIs were not tested, 25 (18.5%) were tested outside the EID guideline, and another 25 (18.5%) were tested per the guideline. On the other hand, among 65 mothers that attended equal or more than four ANC visits, 14 (18.7%) HEIs were not tested, 33 (44.0%) were tested outside the EID guideline, and 21 (32.3%) were tested according to the guideline (*P* < 0.001).

Concerning place of delivery, among 89 mothers that did not deliver in a health facility (hospital, health center, or a health post), 61 (68.5%) HEIs were not tested, 14 (15.7%) were tested outside the EID guideline, and another 14 (15.7%) were tested per the guideline.

Similarly, of 157 mothers that delivered in a health facility, 71 (45.2%) HEIs were not tested, 46 (29.3%) were tested outside the EID guideline, and 40 (25.5%) were tested per the guideline (*P* = 0.002).

Overall, 132 (53.7%; 95% CI: 47.2–60.0) HEIs were not tested for HIV using DNA-PCR (thus EID), 60 (24.4%; 95% CI: 19.1–30.2) were tested outside the EID guideline (early and late), and 54 (21.9%; 95% CI: 16.9–27.6) were tested per the guideline (Table 1).

### 3.2. Factors Associated with Testing of HEIs Outside EID Guideline Compared to Not Testing

In multinomial unadjusted logistic regression (Table 2), testing outside EID guideline relative to no testing increased with maternal age above 30 years (UOR = 2.72; 95% CI: 1.41–5.23; *P* = 0.003), completion of primary (UOR = 0.49; 95% CI: 0.15–1.45; 0.19) and secondary (UOR = 2.72; 95% CI: 1.18–6.29; *P* = 0.019) education, being married (UOR = 2.32; 95% CI: 1.21–4.47; *P* = 0.011), and formal (UOR = 26.32; 95% CI: 5.49–26.16; *P* < 0.001) and self-employment (UOR = 2.23; 95% CI: 1.13–4.40; *P* = 0.02), maternal knowledge of HIV transmission during pregnancy, labor, and delivery, and breastfeeding (UOR = 7.88; 95% CI: 3.72–16.70; *P* < 0.001), maternal knowledge of the correct number of times and schedules of EID (UOR = 6.33; 95% CI: 6.33–15.09; *P* < 0.001), HIV serostatus disclosure (UOR = 6.34; 95% CI: 3.06–13.14; *P* < 0.001), previous attendance of four or more ANC visits (UOR = 8.01; 95% CI: 3.72–17.27; *P* < 0.001), and delivery of the HEI in a health facility: hospital, health center, or health post (UOR = 2.82; 95% CI: 1.42–5.62; *P* = 0.003).

However, it reduced by 47% with more than five-kilometer radius to the health facility (UOR = 0.53; 95% CI: 0.29–0.99; *P* = 0.049) and by 48% with equal or more than 1-hour travel time to reach the health facility on foot (UOR = 0.52; 95% CI: 0.27–0.99; *P* = 0.048).

### 3.3. Factors Associated with Testing of HEIs per EID Guideline Compared to No Testing

In unadjusted multinomial logistic regression analysis (Table 2), DNA-PCR testing per EID guideline relative to no testing increased with maternal age above 30 years (UOR = 3.82; 95% CI: 1.95–7.51; *P* < 0.001), completion of primary (UOR = 1.17; 95% CI: 0.26–5.28; *P* = 0.834) and secondary (UOR = 8.34; 95% CI: 2.34–29.0; *P* = 0.001) education, being married (UOR = 2.42; 95% CI: 1.23–4.74; *P* = 0.001), formal (UOR = 31.82; 95% CI: 6.36–59.18; *P* < 0.001) and self-employment (UOR = 3.42; 95% CI: 1.65–7.10; *P* = 0.001), knowledge of HIV transmission during pregnancy, labor, and delivery (UOR = 10.53; 95% CI: 4.87–22.79; *P* < 0.001), knowledge of the correct number of times and schedules of EID (UOR = 7.42; 95% CI: 3.09–17.84; *P* < 0.001), HIV serostatus disclosure (UOR = 4.61; 95% CI: 2.16–9.83; *P* < 0.001), attendance of equal or more than four ANC visits during recent pregnancy (UOR = 6.80; 95% CI: 3.11–14.85; *P* < 0.001), and delivery of the HEI in a health facility (UOR = 2.54; 95% CI: 1.22–4.93; *P* = 0.012).

Conversely, it decreased by 57% with more than five-kilometer radius distance to the health facility (UOR = 0.43; 95% CI: 0.22–0.82; *P* = 0.011) and by 74% with more than 1-hour travel time to the health facility on foot (UOR = 0.26; 95% CI: 0.13–0.50; *P* < 0.001).

However, two variables in this study, maternal education with VIF of 11.0 and place of delivery of the HEI with VIF of 13.53, were found to be collinear with other variables (see Supplementary Material S1 in Supplementary Material available online at https://doi.org/10.1155/2017/4654763). Consequently, they were excluded from the adjusted analysis.

### 3.4. Adjusted Analysis of Factors Independently Associated with Testing of HEIs

In adjusted multinomial logistic regression analysis (Table 2), testing of HEIs outside EID guideline relative to no testing increased with maternal knowledge of HIV transmission during pregnancy, labor or delivery, and breastfeeding (AOR = 2.90; 95% CI: 1.10–7.65; *P* = 0.032), HIV serostatus disclosure to a spouse, relative or friend or significant others (AOR = 2.70; 95% CI: 1.10–6.63; *P* = 0.030), and four or more ANC visits in the last pregnancy (AOR = 3.25; 95% CI: 1.23–8.59; *P* = 0.017).

In contrast, testing of HEIs per EID guideline relative to no testing increased with maternal age above 30 years (AOR = 2.75; 95% CI: 1.20–6.34; *P* = 0.017) and with maternal knowledge of HIV transmission during pregnancy, labor or delivery, and breastfeeding (AOR = 3.70; 95% CI: 1.39–9.88; *P* = 0.009).

## 4. Discussion

This study investigated the level and factors associated with use of EID among HEIs at Kisenyi HCIV in Kampala district, Uganda. We found slightly more than half of the HEIs were not tested for HIV by DNA-PCR, almost a quarter were tested outside the EID guideline (early and late), and merely 21.9% were tested per the guideline. The proportion of HEIs that received EID by 2 months of age was lower than the standard recommendation of 100% [9] and far below 50% EID use by 8 weeks in 2014 [17]. Evidence from systematic review of 44 studies from 15 countries in sub-Saharan Africa showed that between 48 and 81% of HEIs receive EID within 2 months [18], a result not comparable to level of EID use in the present study. Without timely use of EID, the opportunity for early HIV diagnosis and ART initiation are missed out. It appears timely access to EID is a problem in most sub-Saharan countries. Evidence from a multicenter cohort study in Ethiopia found only 41% of HEIs had EID at 6 weeks and the median age at the time of the testing was 60 days [19]. In Kenya, 43% of HEIs that dropped out of the EID program within 2 months of enrolment never received EID [5], an indication that use of EID reduces with increased dropout rates.

Our study found testing of HEIs by DNA-PCR per EID guideline relative to no testing increased with maternal age above 30 years compared to below 30 years. Earlier in a Kenyan study, young mothers had increased dropout rate of HEIs from the EID program [5]. Differential dropout rates may thus account for EID use. Consistent with our results, an increase in use of maternal health services was reported with increase in maternal age in Nigeria [20].

However, the present finding is contrary to an earlier study that found high use of prevention of MTCT of HIV services among mothers aged 20–30 years that attended antenatal care at Kisenyi and Naguru Health Centers [21].

A probable explanation for the differences in use of EID services between younger and older mothers can be attributed to HIV care experiences developed over time. Mostly, young HIV-positive mothers have poor understanding of HIV transmission and prevention methods compared to older HIV-positive mothers because of their shorter stay in HIV care, treatment, and support programs. Irrespective of maternal age differences in using EID services, all mothers/caregivers of HEIs must be supported by healthcare providers to complete the 18-month follow-up of HEIs [9].

We found maternal knowledge of HIV transmission (during pregnancy, labor or delivery, and breastfeeding) was associated with increased testing of HEIs (per EID guideline and outside the EID guideline). This result highlights the importance of health education in increasing maternal knowledge thus empowering mothers to have an effective control over health determinants. Secondly, it underscores the role of health education in increasing use of available health services. In South Africa, inadequacies in knowledge of MTCT of HIV associated with maternal nonreporting for EID services at 6 weeks [22]. Consequently, HEIs missed DNA-PCR tests at required time points. In Northeastern Uganda, health education talks and counseling of mothers/caregivers of HEIs on the importance of EID significantly improved the use of EID at 6 weeks [23], probably because of enhanced maternal knowledge over determinants of health.

Our result emphasize the importance of maternal knowledge of HIV transmission during pregnancy, labor or delivery, and breastfeeding as a critical catalyst for EID services utilization. In improving utilization of EID services and ensuring HIV-free infant survival, health providers facing similar challenges should focus attention on empowering mothers/caregivers of HEIs on understanding the transmission and prevention of MTCT of HIV. Previously at both Kisenyi and Naguru Heath Centers, good counseling of mothers that attended ANC visits increased use of prevention of MTCT of HIV services [21].

Our study further indicates testing of HEIs by DNA-PCR outside EID guideline increased with maternal HIV serostatus disclosure and attendance of four or more ANC visits during the recent pregnancy. This finding indicates and confirms the importance of ANC in enhancing use of maternal, newborn and child health interventions, and the role of HIV serostatus disclosure in chronic HIV care.

First, disclosure of HIV serostatus is critical in accessing existing HIV prevention, care, treatment, and support services and in galvanizing family, spousal, friend, and relative support. This finding hence confirms the importance of HIV serostatus disclosure in general. With some HIV-positive mothers still having undisclosed HIV serostatus, health workers in EID programs should strengthen efforts in helping these mothers in disclosing their HIV serostatus (to someone they can confide in). This will assure them access to emotional, psychological, social, and medical support without hindrance. At enrolment of HIV-positive mothers into HIV care, and during adherence preparation for ART, the benefits of serostatus disclosure are further explained.

In PHDP (Positive Health, Dignity, and Prevention), a set of HIV prevention interventions that aims at keeping people living with HIV physically, mentally, and psychologically healthy, disclosure is a key component.

Second, in Uganda, all pregnant women are required to attend at least four ANC visits during which they receive routine HIV testing with same day results, and those testing HIV-positive are supported to disclosure their serostatus. During ANC visits, all pregnant women receive health education talks on mode and prevention of mother to child transmission of HIV among others. The factors explained above might have accounted for the increased EID by DNA-PCR testing among HIV-positive mothers with disclosed serostatus [24]. However, it is surprising that attendance of four or more ANC visits did not significantly increase testing of HEIs by DNA-PCR per EID guideline, suggesting the need to continuously support HIV-positive mothers in understanding the number, schedule, and importance of EID. This is because early testing results in most HIV-positive cases being missed because of low sensitivity of DNA-PCR test before 6 weeks. Studies indicate that HIV-DNA PCR assays identify between 20% and 55% of HIV-positivity among HEIs from birth through the first week of life, but this increases to more than 90% by 2–4 weeks of age and to 100% at 3–6 months [25]. So by testing early, there is a risk of missed HIV diagnosis. Contrariwise, late testing results in late HIV diagnosis, late ART initiation, and worsened HIV morbidity and mortality.

## 5. Study Limitation

Although this study underscored critical factors associated with use of EID, the findings should be interpreted while considering certain limitations. First, we did not examine the technical competence of staffs and their availability, health facility waiting times, maternal reason(s) for choice of EID services, interpersonal relations with healthcare providers, and mode of transport among others as factors that might influence EID use. However, our results provide additional evidence for healthcare providers, healthcare managers, researchers, and other public health professionals in improving delivery of EID services in Kampala, Uganda.

## 6. Conclusion

A substantial number of HEIs were not tested by DNA-PCR, many were tested either early or late, and few were tested per EID guideline. Testing of HEIs per EID guideline was associated with maternal age above 30 years; testing outside EID guideline was associated with maternal HIV serostatus disclosure and equal or more than four ANC visits. However, maternal knowledge of HIV transmission (during pregnancy, labor or delivery, and breastfeeding) was associated with testing of HEIs per EID guideline and outside EID guideline.

## 7. Recommendation

In order to increase use of EID, improvement in maternal knowledge of EID is needed particularly on the importance of EID, the frequency, and schedule during ANC visits; the benefits of HIV serostatus disclosure so as to galvanize social, psychological, and medical support from the spouse, relatives, family members, friends, or significant others are needed. This is achievable through health education or routine counseling. In addition, EID programs should recognize maternal age differences as a key factor in use of EID services.

## Supplementary Material

Collinear variables excluded from multivariate model.

## Figures and Tables

**Table 1:** Sociodemographic characteristics of respondents and use of EID.

	HEIs not tested by DNA-PCR	HEIs tested by DNA-PCR outside EID guideline	HEIs tested by DNA-PCR per EID guideline	Total	*P* value
Overall	132 (53.7%)	60 (24.4%)	54 (21.9%)	246 (100.0%)	
Maternal age (years)					<0.0001
Less than 30 years	103 (63.2)	34 (20.9)	26 (16.0)	163	
More than 30 years	29 (34.9)	26 (31.3)	18 (33.7)	83	
Mean ± SD	25.5 ± 6.12	29.3 ± 5.35	29.7 ± 5.69	246	0.0003
Maternal education					<0.001
None	31 (72.1)	9 (20.9)	3 (7.0)	43	
Primary	44 (80.0)	6 (10.9)	5 (9.1)	55	
Secondary and over	57 (38.5)	45 (30.4)	46 (31.1)	148	
Marital status					0.008
Single	101 (60.5)	35 (21.0)	31 (18.6)	67	
Married	31 (39.2)	25 (31.7)	23 (29.1)	79	
Form of employment					<0.001
None	81 (70.4)	20 (17.4)	14 (12.2)	115	
Formal	2 (7.7)	13 (50.0)	11 (42.3)	26	
self	49 (46.7)	27 (25.7)	29 (27.6)	105	
Mother knows that HIV is transmissible during pregnancy, labor and delivery, and breastfeeding					<0.001
No	118 (68.2)	31 (17.9)	24 (13.9)	173	
Yes	14 (19.2)	29 (39.7)	30 (41.1)	73	
Mother knows the correct frequency and timing of all HIV testing in HEIs					<0.001
No	123 (61.8)	41 (20.6)	35 (17.6)	199	
Yes	9 (19.2)	19 (40.4)	19 (40.4)	47	
Disclosed HIV status					<0.001
No	116 (64.1)	32 (17.7)	33 (18.2)	181	
Yes	16 (24.6)	28 (43.1)	21 (32.3)	65	
Number of ANC visits					<0.001
Less than four	85 (63.0)	25 (18.5)	25 (18.5)	135	
Four or more	14 (18.7)	33 (44.0)	28 (37.3)	75	
Place of delivery					0.002
Outside health facility	61 (68.5)	14 (15.7)	14 (15.7)	89	
In a health facility	71 (45.2)	46 (29.3)	40 (25.5)	157	

**Table 2:** Factors associated with use of EID among HEIs, Kisenyi HC IV, Kampala, Uganda.

Characteristics	Unadjusted multinomial logistic regression analysis				Adjusted multinomial logistic regression analysis			
	HEI tested by DNA-PCR outside EID guideline versus HEI not tested	*P* value	HEI tested by DNA-PCR per EID guideline versus HEI not tested	*P* value	HEI tested by DNA-PCR outside EID guideline versus HEI not tested	*P* value	HEI tested by DNA-PCR per EID guideline versus HEI not tested	*P* value
	UOR (95% CI)		UOR (95% CI)		AOR (95% CI)		AOR (95% CI)	
Maternal age								
≤30 years	1		1		1		1	
>30 years	2.72 (1.41–5.23)	0.003	3.82 (1.95–7.51)	<0.001	1.97 (0.87–4.49)	0.106	2.75 (1.20–6.34)	**0.017**
Marital status								
Single	1		1		1		1	
Married	2.32 (1.21–4.47)	0.011	2.42 (1.23–4.74)	0.01	1.28 (0.55–2.98)	0.57	1.35 (0.57–3.15)	0.494
Form of employment								
None	1		1		1		1	
Formal	26.32 (5.49–26.16)	<0.001	31.82 (6.36–9.18)	<0.001	7.54 (0.77–5.89)	0.082	7.68 (0.75–8.99)	0.086
self	2.23 (1.13–4.40)	0.02	3.42 (1.65–7.10)	0.001	1.19 (0.5–2.77)	0.691	1.67 (0.68–4.08)	0.265
Mother knows HIV is transmissible at pregnancy, labor and delivery, and breastfeeding								
No	1		1		1		1	
Yes	7.88 (3.72–16.70)	<0.001	10.53 (4.87–22.79)	<0.001	2.90 (1.10–7.65)	**0.032**	3.70 (1.39–9.88)	**0.009**
Mother knows the correct frequency and timing of EID								
No	1		1		1		1	
Yes	6.33 (2.66–15.09)	<0.001	7.42 (3.09–17.84)	<0.001	1.12 (0.36–3.54)	0.846	1.61 (0.50–5.15)	0.425
Disclosed HIV serostatus								
No	1		1		1		1	
Yes	6.34 (3.06–13.14)	<0.001	4.61 (2.16–9.83)	<0.001	2.70 (1.10–6.63)	**0.030**	1.60 (0.62–4.41)	0.332
ANC visits								
<4 visits	1		1		1		1	
≥4 visits	8.01 (3.72–17.27)	<0.001	6.80 (3.11–14.85)	<0.001	3.25 (1.23–8.59)	**0.017**	2.45 (0.88–6.81)	0.086
Distance to the health facility								
≤5	1		1		1		1	
>5	0.53 (0.29–0.99)	0.049	0.43 (0.22–0.82)	0.011	0.87 (0.26–2.95)	0.825	1.81 (0.45–7.28)	0.400
Time to health facility								
<1 hour	1		1		1		1	
≥1 hour	0.52 (0.27–0.99)	0.048	0.26 (0.13–0.50)	<0.001	1.15 (0.33–3.98)	0.829	0.35 (0.09–1.42)	0.142

*Note*. AOR: adjusted odds ratio; *P* < 5% considered statistically significant; UOR: unadjusted odds ratio; variables with significant correlation with other variables (VIF > 10) were excluded.

## Abbreviations

ANC: Antenatal care
AOR: Adjusted odds ratio
ART: Antiretroviral therapy
DNA-PCR: Deoxyribonucleic Acid-Polymerase Chain Reaction
EID: Early infant diagnosis of HIV
EPNC: Early postnatal-care
HC: Health center
HEI: HIV exposed infant
HIV: Human immunodeficiency virus
MTCT: Mother to child transmission
PHDP: Positive Health, Dignity, and Prevention
PNC: Postnatal-care
UOR: Unadjusted odds ratio
WHO: World Health Organization.

## Data Access

The data shall not be shared in order to protect participant's anonymity.
