# Supplementary material for: Early Diagnosis of HIV among Infants Born to HIV-Positive Mothers on Option-B Plus in Kampala, Uganda
Source: AIDS Res Treat. 2017 Oct 19;2017:4654763. doi: 10.1155/2017/4654763 (PMC5671668; doi:10.1155/2017/4654763)
Supplement: Supplementary file 1 — Collinear variables excluded from multivariate model. [file 4654763.f1.pdf]

Supplementary material S1: Collinear variables in the model

	**Unadjusted multinominal logistic regression analysis**			
Characteristics	HEI tested by DNA-PCR outside EID guideline vs HEI not tested		HEI tested by DNA-PCR per EID guideline vs HEI not tested	
	UOR (95% CI)	P-value	UOR (95% CI)	P-value
Maternal education				
None	1		1	
Primary	0.49 (0.15-1.45)	0.190	1.17 (0.26-5.28)	0.834
Secondary and over	2.72 (1.18 -6.29)	0.019	8.34 (2.34-29.0)	0.001
Health facility delivery				
No	1		1	
Yes	2.82 (1.42-5.62)	0.003	2.54 (1.22-4.93)	0.012
